# Single-cell RNA transcriptomics reveals differences in the immune status of alcoholic and hepatitis B virus-related liver cirrhosis

**DOI:** 10.3389/fendo.2023.1132085

**Published:** 2023-02-02

**Authors:** Pengpeng Zhang, Hao Li, Bo Peng, Yu Zhang, Kai Liu, Ke Cheng, Yingzi Ming

**Affiliations:** ^1^ The Transplantation Center of the Third Xiangya Hospital, Central South University, Changsha, Hunan, China; ^2^ Engineering & Technology Research Center for Transplantation Medicine of National Ministry of Health, Changsha, Hunan, China

**Keywords:** alcoholic liver cirrhosis, HBV-related liver cirrhosis, scRNA-seq, galectin-9, lipid metabolism

## Abstract

**Background:**

Alcoholic and hepatitis B virus (HBV)-related liver cirrhosis has placed a tremendous burden on the healthcare system with limited treatment options. This study explored the differences in the immune status of alcoholic and HBV-related liver cirrhosis.

**Methods:**

A total of 15 human liver samples from the Third Xiangya Hospital of Central South University, including five healthy controls (HC group), five alcoholic cirrhosis patients (ALC group), and five HBV-related cirrhosis patients (HBV group) were used. Of these, eight samples, including 3 HC group, 2 ALC group and 3 HBV group, were randomly collected to do single-cell RNA sequencing (scRNA-seq). The degree of steatosis was assessed by H&E staining and the presence of intrahepatic immune cells was evaluated by immunochemistry (IHC).

**Results:**

The immune status of alcoholic and HBV-related liver cirrhosis differed significantly. ScRNA-seq analysis identified a higher ratio of intrahepatic monocyte/macrophages and an obvious decreased ratio of T cells and B cells in the ALC group than in the HBV group. IHC staining of intrahepatic monocyte/macrophages, T and B cell exhibited similar results with scRNA-seq analysis. CD5L^+^ Kupffer cells, a cell type involved in lipid metabolism, were the major monocyte/macrophage subset in ALC liver tissue. H&E staining indicated that the level of steatosis was more severe in the ALC than in the HBV group. Ligand/receptor analysis showed that the T cell exhaustion observed in the ALC liver may be related to the expression of Galectin-9 on Kupffer cells. Fewer B cells were also found in the ALC group and most had higher lipid metabolism, reduced ribosomal activity, and a dysregulated mitochondrial oxidative phosphorylation system. Moreover, scRNA-seq showed a significantly lower ratio of plasma B cells, indicating that the humoral immune response in the ALC liver was similarly dysfunctional. Ligand/receptor analysis also discovered that Galectin-9 expressed on Kupffer cells may inhibit humoral immunity.

**Conclusion:**

Patients with ALC have different immune characteristics than those with HBV-induced cirrhosis, including an increased ratio of intrahepatic monocyte/macrophages and a dysfunctional adaptive immune response in the liver. Galectin-9 could serve as a potential therapeutic target for ALC treatment.

## 1 Introduction

Liver cirrhosis, the end stage of progressive liver fibrosis, is estimated to affect 1–2% of people and contribute to >1 million deaths globally each year (1, 2). This condition can be caused by chronic injury arising from alcohol abuse and hepatitis. Once decompensation occurs, there is a rapid progression to acute-on-chronic liver failure (ACLF), a syndrome with a high short-term mortality rate that puts a tremendous burden on the healthcare system. The main cause of cirrhosis in developed countries, including China, are HBV infection, followed by alcohol misuse (1, 2). A recent study in northern China found that the incidence of HBV-related liver failure has gradually decreased while alcoholic liver cirrhosis (ALC) has increased over the past 10 years (3). In 2010, ALC was globally responsible for around 493,300 (0.9%) of all global deaths (4). These findings indicate that new and effective methods are urgently needed to reduce ALC-associated incidence and mortality rates.

Liver cirrhosis is a diffuse pathophysiological state of the liver, characterized by chronic necroinflammatory and fibrogenic processes that convert the normal liver architecture into structurally abnormal nodules, dense fibrotic septa, and concomitant parenchymal exhaust, causing the eventual collapse of the liver tissue (5). Active hepatic inflammation involving both innate and adaptive immune regulation plays a pivotal role in the inflammation-necrosis-regeneration process that eventually leads to liver cirrhosis (5, 6). Macrophages are the primary source of hepatic inflammation during ALC (7, 8). When mice are exposed to ethanol, the Ly6C^high^/Ly6C^low^ ratio increases, leading to significant liver injury. However, upon phagocytosis of apoptotic hepatocytes, pro-inflammatory Ly6C^high^ monocytes can switch to anti-inflammatory Ly6C^low^ cells, suggesting that macrophages play a complex role in ALC pathogenesis (9). Macrophages can also be activated by high-titer HBV to produce inflammatory cytokines such as TNF-α and IL-6 and mount a virus-specific immune response (10). Adaptive immune cells also regulate the ALC process, including an alcohol-induced reduction in peripheral T and B cell numbers (11, 12). Thus, the characteristics of the immune response may be critical to the development of liver cirrhosis. An in-depth investigation of the immune status of alcoholic and HBV-related liver cirrhosis could provide a solid theoretical foundation to better understand the pathophysiology of these conditions.

Single-cell RNA sequencing (scRNA-seq) technology assesses the transcriptome at the single-cell level and evaluates transcriptional differences and similarities between cell populations (13). With its extremely high resolution, this technology can distinguish cell heterogeneity, and explore and define rare cell subgroups and their role in immune-related diseases (14). ScRNA-seq can also be used to predict key regulatory transcription factors (14). Our recent study used scRNA-seq to identify the status of hepatic lymphatic endothelial cells in HBV-related ACLF, informing the mechanism of this disease (15). Iwakiri et al. revealed zone-specific alterations of liver sinusoidal endothelial cells in liver cirrhosis using scRNA-seq (16). Based on the advantages of scRNA-seq, the present study explored the immune status of alcoholic and HBV-related liver cirrhosis. We found that patients with ALC had different immune characteristics than those with HBV-induced cirrhosis, including an increased ratio of intrahepatic monocyte/macrophages and a dysfunctional adaptive immune response in the liver, which could be linked to the expression of Galectin-9 on macrophages.

## 2 Methods

### 2.1 Study population and collection of human liver samples

The present study included 15 human liver samples which referred to 5 health control (HC group), 5 HBV-related cirrhosis patients (HBV group) and 5 alcoholic cirrhosis patients (ALC group). Among them, 8 liver samples, including 3 HC group, 2 ALC group and 3 HBV group, were randomly collected to do scRNA-seq. HC liver samples were derived from cardiac death donors. All cirrhosis liver samples were collected from the patients who underwent liver transplant.

### 2.2 Ethics approval and consent to participants

Written informed consent was obtained from all participants, and this study was approved by the Ethics Committee of The Third Xiangya Hospital Central South University (No:2020-S024). At the same time, all methods were performed in accordance with the ethical guidelines and regulations of the 1975 Declaration of Helsiniki.

### 2.3 Liver tissue dissociation and preparation

The fresh liver tissues were stored in the GEXSCOPE^®^ Tissue Preservation Solution (Singleron) and transported to the Singleron lab on ice as soon as possible. The specimens were washed with Hanks Balanced Salt Solution (HBSS) for 3 times and minced into 1–2 mm pieces. Then the tissue pieces were digested with 2ml GEXSCOPE^®^ Tissue Dissociation Solution (Singleron) at 37°C for 15min in 15ml centrifuge tube with sustained agitation. After digestion, we used 70 um sterile strainers to filter the samples and centrifuged the samples at 50g for 5 minutes to remove hepatocyte (17). Then the precipitation was discarded, and the supernatant was resuspended in 1ml PBS (HyClone). Centrifuging the samples at 300g for 5 minutes, then the supernatant was discarded, and the precipitation was resuspended in 1ml PBS (HyClone). To remove the red blood cells, 2 mL GEXSCOPE^®^ red blood cell lysis buffer (Singleron) was added for 10 minutes at 25°C. The solution was then centrifuged at 500g for 5 min and suspended in PBS. The sample was stained with trypan blue (Sigma) and microscopically evaluated. Then those cells were almost liver non-parenchymal cells (NPCs) with less hepatocytes.

### 2.4 ScRNA-seq and primary analysis of raw read data

Single-cell suspensions with 1×10^5^ NPC cells/mL concentration in PBS (HyClone) were prepared. Single-cell suspensions were then loaded onto microfluidic devices and scRNA-seq libraries were constructed according to Singleron GEXSCOPE^®^ protocol by GEXSCOPE^®^ Single-Cell RNA Library Kit (Singleron Biotechnologies). Individual libraries were diluted to 4nM and pooled for sequencing. Pools were sequenced on Illumina HiSeq X with 150 bp paired end reads. Raw reads were processed with fastQC and fastp to remove low quality reads. Poly-A tails and adaptor sequences were removed by cutadapt. After quality control, reads were mapped to the reference genome GRCh38 (ensembl version 92 annotation) using STAR. Gene counts and UMI counts were acquired by featureCounts software. Expression matrix files for subsequent analyses were generated based on gene counts and UMI counts.

### 2.5 Quality control, dimension-reduction, and clustering

Cells were filtered by gene counts greater than 200 and UMI counts below 6,000. Cells with over 25% mitochondrial content were removed. After filtering, 39345 cells were retained for the downstream analyses. We used functions from Seurat v4.0 for dimension-reduction and clustering (18).

### 2.6 Genes and Genomes pathway enrichment analysis

To investigate the potential functions of immune cells clusters, Genes and Genomes pathway enrichment analysis were used with the Metascape (19).

### 2.7 Ligand/receptor analysis

To investigate the potential interaction between endothelial/epithelial clusters and macrophage clusters, Ligand/receptor analysis were used with the “iTalk” and “CellChat” R package version.

### 2.8 Immumohistochemical staining

Liver samples were fixed in 10% neutralized buffered formaldehyde at 4 °C for 48 hours. Paraffin blocks were made and 4 μm slides were deparaffinized with xylenes and rehydrated by washing through a graded alcohol series to deionized water. The hydrated tissue sections were washed with phosphate-buffered saline (PBS). To retrieve antigens, the sections were incubated with 10 mM EDTA buffer (pH 6.0) for 15 min at approximately 100 °C. After the primary antibody was incubated overnight at 4°C, the excess primary antibody was washed away with PBS, the non-specific antigen was blocked by the catalase enzyme body for 20 minutes, and the secondary antibody was incubated for 30 minutes for color development. The target antigen was developed by DAB horseradish peroxidase color development methods (Kit-0015, MXB, China).

### 2.9 Hematoxylin and eosin staining

Paraffin sections were deparaffinized with xylenes and rehydrated by washing through a graded alcohol series to deionized water. The sections were stained by hematoxylin for 5 minutes and eosin for 10 minutes, and washed by warm water for 5 minutes. Then the sections were dehydrated by washing through a graded alcohol series to xylenes and mounted with neutral resins (20200206, YiYang, China).

### 2.10 Image acquisition and quantification

Images were obtained using Leica Microsystems DMI 3000B observer. For CD68, CD3, CD19 and steatosis area quantification, at least 10 images X100 magnification were obtained per slide. The number of positive cells and steatosis area were measured by Image J (NIH) and subjected to statistical analysis.

### 2.11 Statistical analysis

All data were expressed as mean ± standard error of mean (SEM). The comparison between two groups was performed using unpaired Student’s t-test. The comparison between multiple groups was performed using one-way ANOVA. The P-value less than 0.05 was considered statistically significant.

## 3 Results

### 3.1 ScRNA-seq revealed differences in the immune status of liver tissue in the HC, ALC, and HBV groups

**Figure 1:**
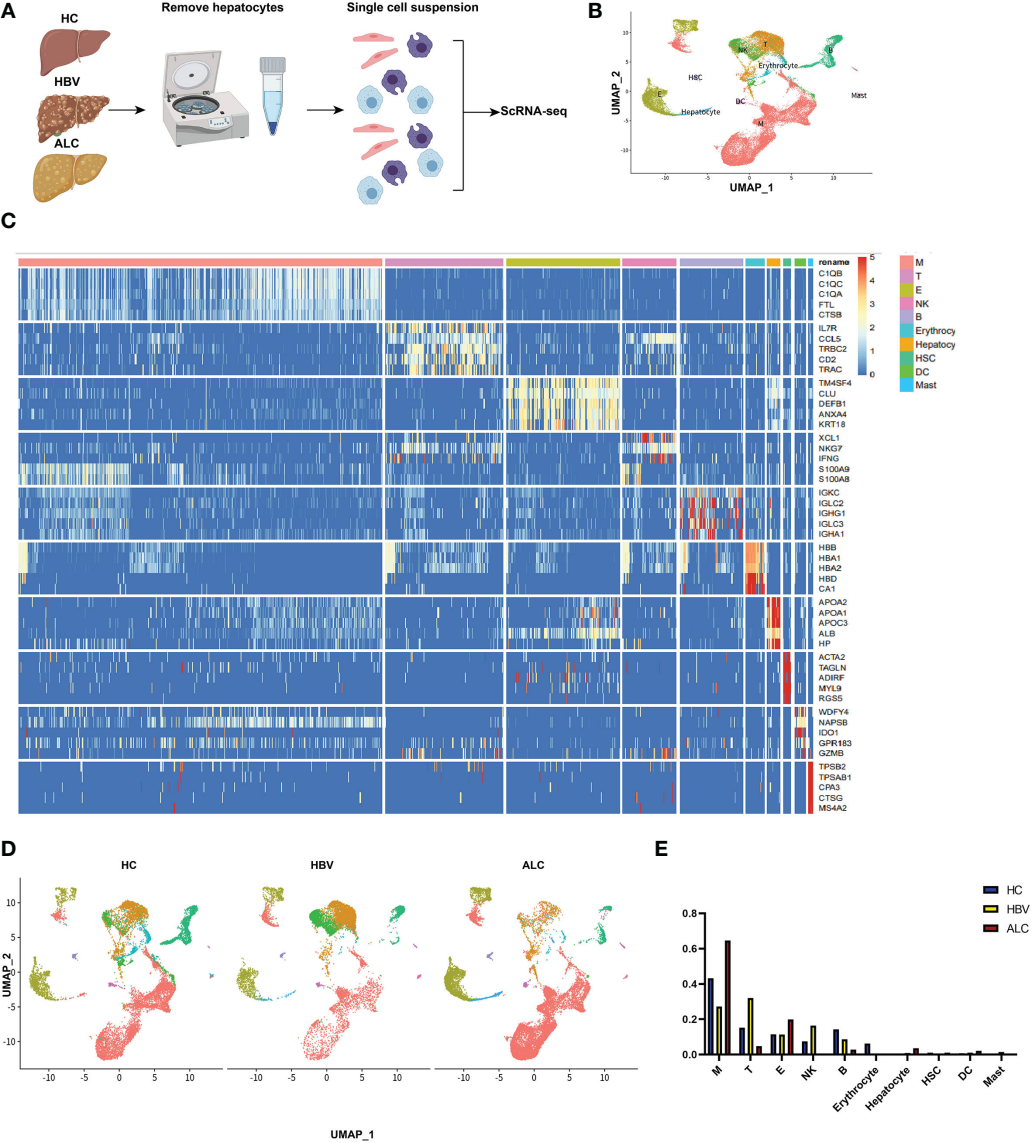
scRNA-seq identified the characteristics of immune cells among three groups. **(A)** The workflow of scRNA-seq analysis. **(B)** 39,345 NPCs from HC (n=3), ALC (n=2) and HBV (n=3) liver tissues were annotated into 10 clusters and shown on a UMAP plotting. M: monocyte/macrophage; T: T cell; E, endothelial/epithelial cell; NK, natural killer cell; B, B cell; HSC, hepatic stellate cell; DC, dendritic cell. **(C)** A heatmap of the high-expression genes in the 10 subpopulations. **(D)** A UMAP plotting of the 10 classical cluster distribution of each group. **(E)** The proportion of 10 classical clusters in the HC, HBV, and ALC groups.

**Figure 2:**
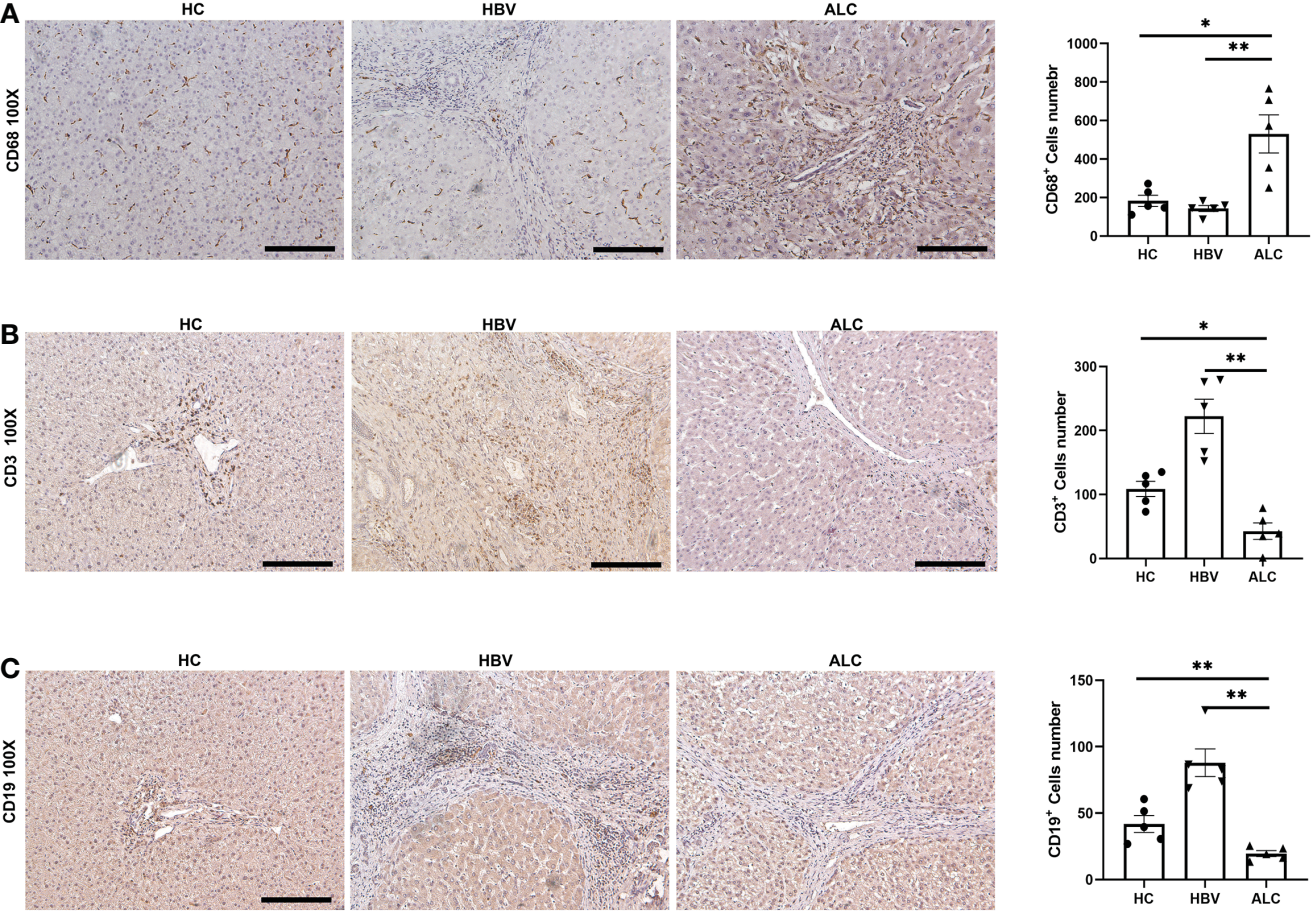
The IHC staining of monocyte/macrophage, T cells and B cells among three groups. **(A)** IHC staining of CD68^+^ monocyte/macrophages in liver tissues. **(B)** IHC staining of CD3^+^ T cells in liver tissues. **(C)** IHC staining of CD19^+^ B cells in liver tissues (*p <0.05, **p <0.01, 100x, bar = 200μm).

To characterize the immune status of patients with HC, ALC, and HBV-related cirrhosis, scRNA-seq was performed on NPCs isolated from liver tissue samples from the HC (n=3), ALC (n=2), and HBV (n=3) groups. The clinical data of these patients in ALC and HBV group was summarized in **Table S1**. Most hepatocytes were excluded by low-speed centrifugation and only NPCs isolated from liver tissues were subjected to the following scRNA-seq analysis (**Figure 1A**). Quality control for each sample was done by assessing viability, RNA count, UMI count and mitochondrial gene ratio (**Figure S1**, **Figure S2**). After removing the suspicious double and low-activity cells, 13,212 cells from the HC group, 15,761 from the ALC group, and 10,372 cells from the HBV group were obtained for further analysis. The cells were re-clustered and annotated as monocyte/macrophage (M), T cell (T), endothelial/epithelial cells(E), B cell (B), natural killer cell (NK), erythrocyte, hepatocyte, hepatic stellate cell (HSC), dendritic cell (DC) and mast cell (Mast) subpopulations (**Figure 1B**). The classical marker genes of each subpopulation were shown in **Figures S3**, **S4** and the representative genes of these subpopulations were presented as in a heatmap (**Figure 1C**). UMAP plotting revealed significant differences in the immune cell subpopulations of each group (**Figure 1D**). The ratio of the ten subpopulations was also assessed (**Figure 1E**). The number of monocyte/macrophages was significantly higher and the numbers of T and B cells were lower in the ALC group than in the HBV group. IHC was used to determine the numbers of CD68^+^ monocyte/macrophages (**Figure 2A**), CD3^+^ T cells (**Figure 2B**), and CD19^+^ B cells (**Figure 2C**) in the three groups. The IHC results were consistent with scRNA-seq data, indicating that the scRNA-seq analysis was reliable. In summary, there was a significant difference in the innate and adaptive immune responses associated with alcoholic and HBV-related liver cirrhosis.

### 3.2 The characteristics of intrahepatic monocyte/macrophages associated with ALC and HBV-related cirrhosis

**Figure 3:**
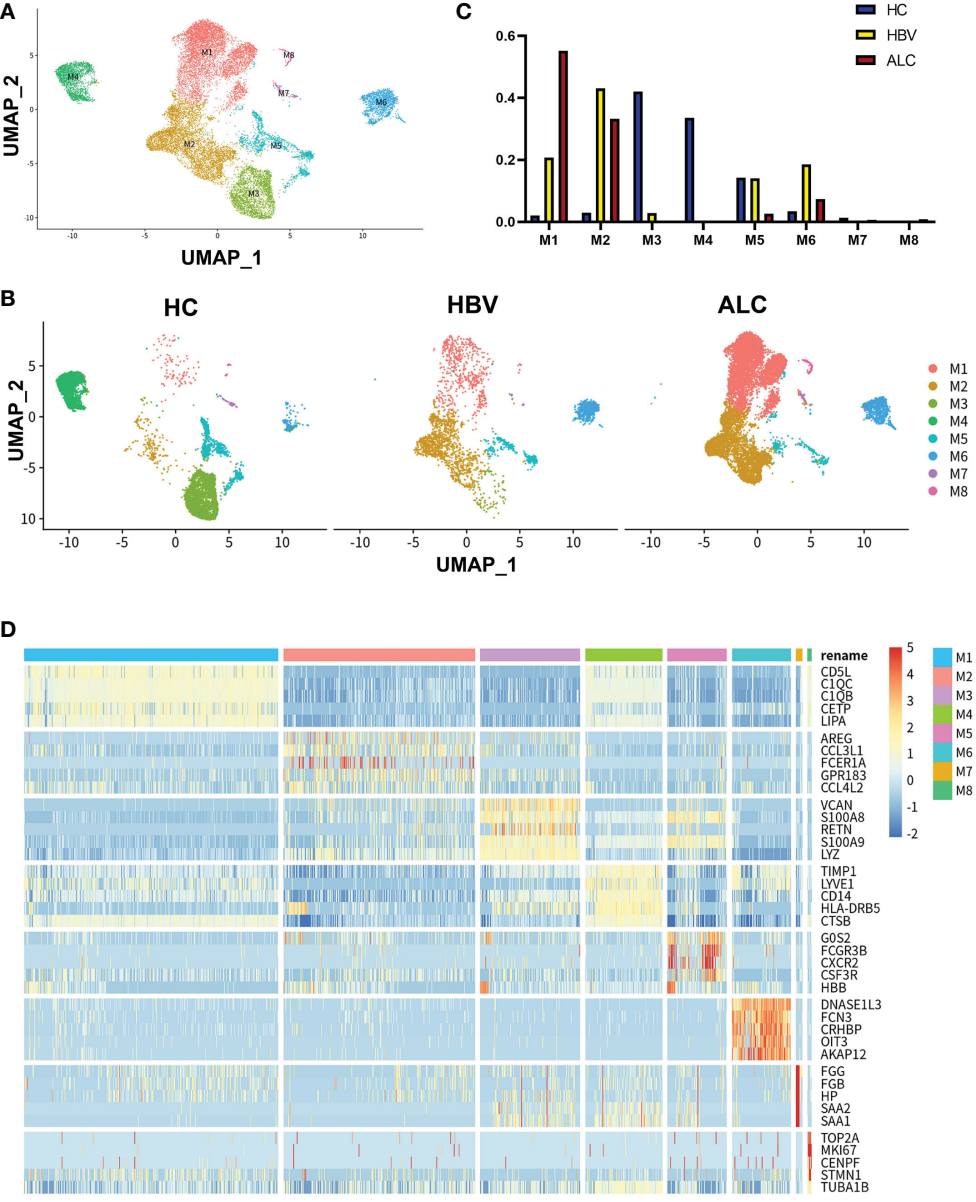
scRNA-seq identified the characteristics of monocyte/macrophages among three groups. **(A)** A total of 18,718 monocyte/macrophages from the HC, ALC, and HBV groups were classified into M1–M8 clusters **(B)** The distribution of the M1–M8 clusters in each group were shown using a UMAP plotting. **(C)** The proportion of the M1–M8 clusters in patients from the HC, HBV, and ALC groups. **(D)** High-expression genes of the M1–M8 clusters were presented using a heatmap.

**Figure 4:**
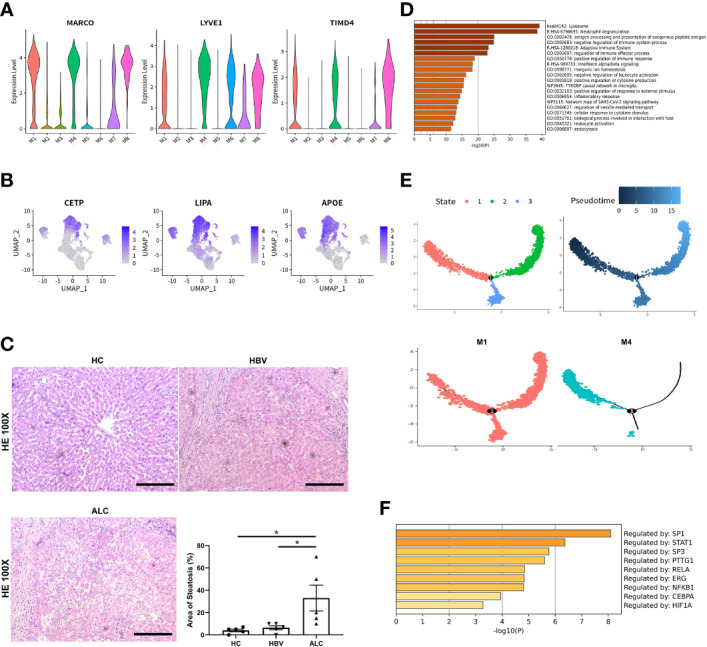
The origin and function of M1–M8 clusters. **(A)** The expression of hepatic resident marker genes in M1–M8 clusters was shown using a violin plotting. **(B)** The expression of lipid metabolism-related genes M1–M8 clusters was shown using a UMAP plotting. **(C)** H&E staining of liver tissue in the HC, HBV, and ALC groups to assess the steatosis (100x). **(D)** Enrichment of high-expression genes in the M1 clusters. **(E)** The Pseudotime trajectory analysis of M1 and M4 clusters. **(F)** Transcription factor analysis of highly expressed genes at the branch point in the Pseudotime trajectory analysis (*p <0.05, bar = 200μm).

To compare the characteristics of intrahepatic monocyte/macrophages in the ALC and HBV groups, the monocyte/macrophage subpopulations were further analyzed using scRNA-seq. A total of 18,718 monocyte/macrophages from the HC, ALC, and HBV groups were classified into M1–M8 clusters (**Figure 3A**). The proportions of M1–M8 clusters differed considerably among the three groups (**Figures 3B, C**). In addition, the heatmap showed that the M1–M8 clusters had significantly different representative marker genes, suggesting that the cluster analysis was specific (**Figure 3D**). The high ratio of monocyte/macrophages in the ALC group suggested that the M1 cluster may play an important role in the innate immune response in ALC patients. Violin plotting showed that the tissue-resident marker genes, MARCO, LYVE l, and TIMD 4, were highly expressed on M1 cells, indicating that the M1 cluster may be Kupffer cells (KCs) (**Figure 4A**). Meanwhile, this cluster also had high expression of lipid metabolism-associated genes, such as CETP, LIPA, and APOE (**Figure 4B**). The level of steatosis was also assessed using H&E staining. The ALC group had a greater degree of steatosis than the HBV group (**Figure 4C**).

Functionally, high-expression gene enrichment analysis showed that the M1 cluster was involved in negatively regulating immune processes (**Figure 4D**), which may inhibit the response to pathogens in ALC patients. In addition, both the M4 and M5 monocyte/macrophage clusters, which actively defend against pathogens (**Figures S5**, **S6**), were reduced in the ALC group (**Figure 3C**). Moreover, the heatmap showed that CD5L was the representative marker gene of the M1 cluster (**Figure 3D**). Finally, Pseudotime trajectory analysis revealed that cells in the M4 cluster (the primary monocyte/macrophages in the HC group) differentiated directly into cells in the M1 cluster (the primary monocyte/macrophages in the ALC group) (**Figure 4E**). Transcription factor analysis of highly expressed genes at the branch point of this trajectory was performed to identify key pathways. SP1 was shown to play a key role in the differentiated trajectory (**Figure 4F**).

### 3.3 The characteristics of intrahepatic T cells associated with ALC and HBV-related cirrhosis

**Figure 5:**
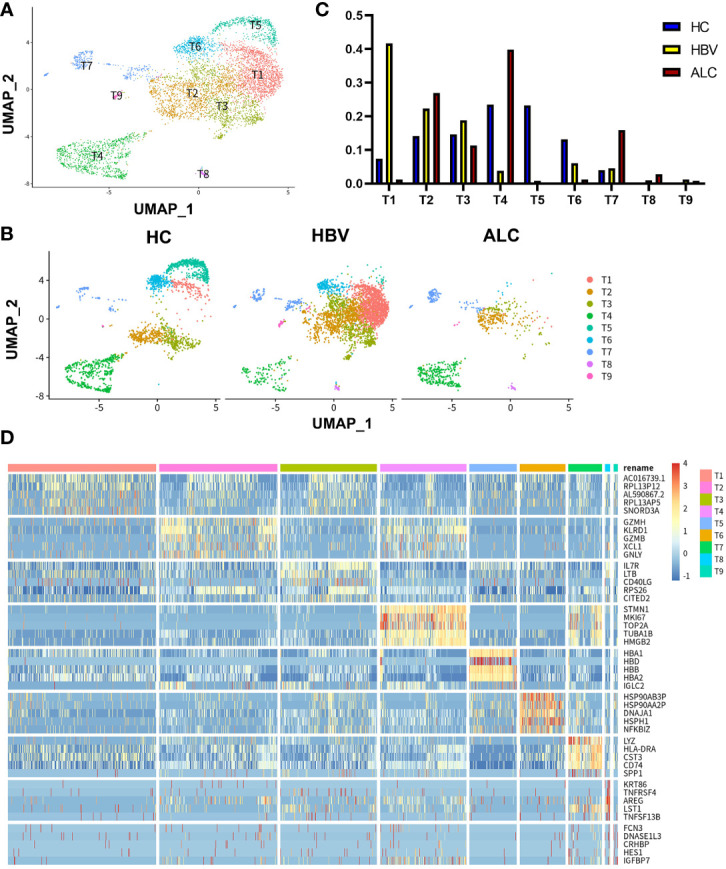
scRNA-seq identified the characteristics of T cells among three groups. **(A)** A total of 6,074 T cells from the HC, ALC, and HBV groups were classified into T1–T9 clusters. **(B)** The distribution of the T1-T9 clusters in each group was shown using a UMAP plotting. **(C)** The proportion of the T1-T9 clusters in patients from the HC, HBV, and ALC groups. **(D)** High-expression genes of the T1-T9 clusters were presented using a heatmap.

**Figure 6:**
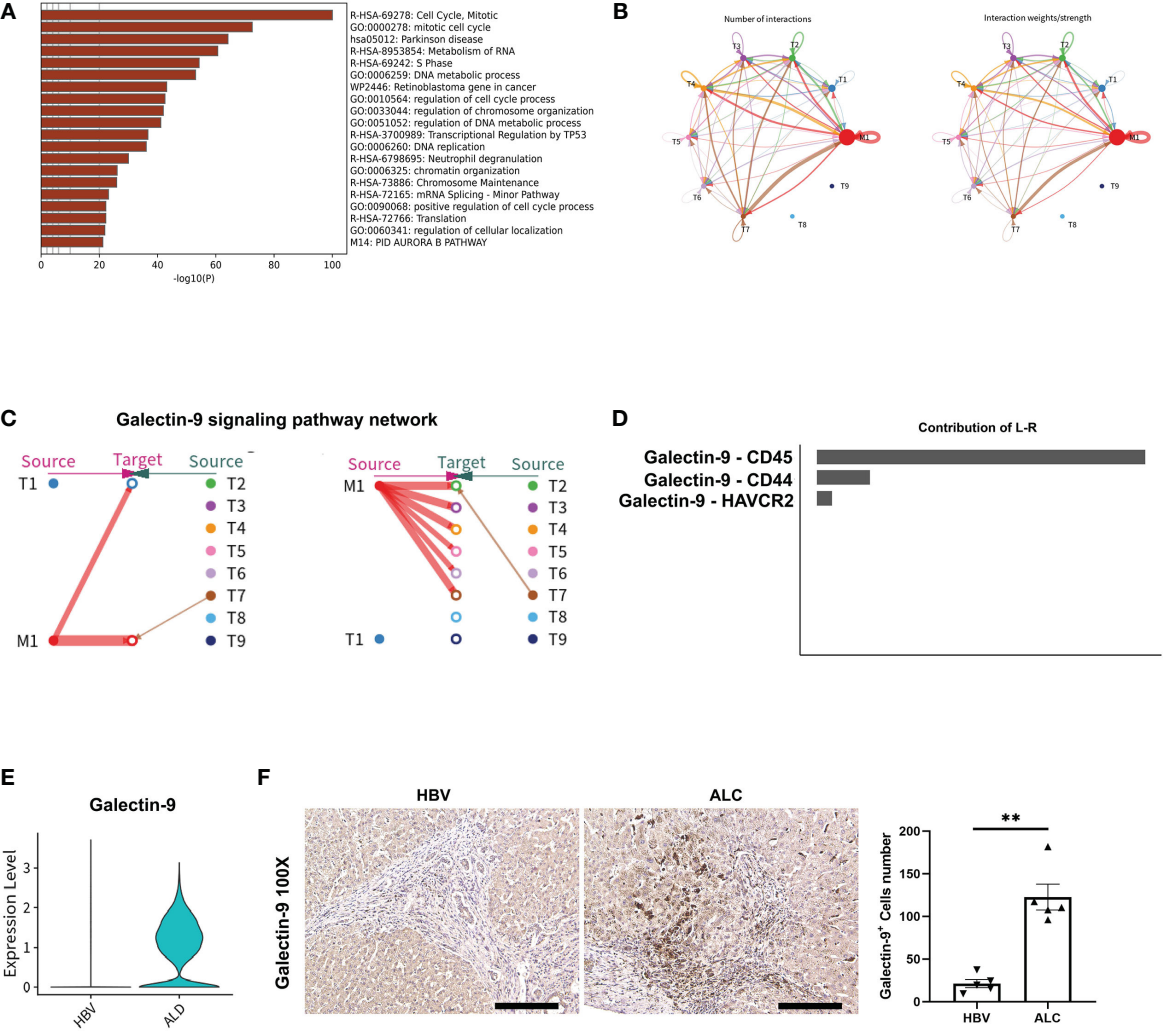
The potential regulatory pathway between M1 and T cells. **(A)** Enrichment of the highly expressed genes in the T4 clusters. **(B)** The Ligand/receptor analysis between the M1 and T1–T9 clusters. **(C)** Galectin-9 was identified as a potential signaling pathway and Galectin-9 was mainly originated from the M1 cluster. **(D)** Galectin-9-CD45 was the mainly Ligand-receptor pathway. **(E)** Galectin-9 expression in liver tissue from the HBV and ALC groups as shown using a violin plot. **(F)** IHC staining for Galectin-9 in the ALC and HBV groups. (**p <0.01, 100x, bar = 200μm).

To compare the characteristics of intrahepatic T cells associated with ALC and HBV-related cirrhosis, 6,074 T cells (CD3^+^) from the HC, ALC, and HBV groups were analyzed and annotated into nine clusters (T1–T9, **Figure 5A**). The proportions of the T1–T9 clusters differed considerably among the three groups (**Figures 5B, C**) and the T4 cluster had a significantly higher ratio in the ALC group than in the HBV group. The heatmap showed that cells in the T4 cluster exhibited a typical proliferation phenotype (**Figure 5D**). Moreover, high-expression gene enrichment analysis found that cells in this cluster were closely related to the cell cycle (**Figure 6A**), implying that the lower number of T cells in the ALC group did not result from inhibited proliferation. Instead, it was hypothesized that the M1 cluster, the primary monocyte/macrophage subset in the ALC group, contributed to the T cell deficiency. Subsequently, Ligand/receptor analysis identified multiple interactions between the M1 cluster and T cells (**Figure 6B**). Galectin-9 was identified as a potential signaling pathway (**Figure 6C**), primarily through its interaction with the CD45 receptor (**Figure 6D**). Further analysis revealed that Galectin-9 was mainly originated from the M1 cluster (**Figure 6C**). Both violin plotting and IHC staining showed that Galectin-9 expression was significantly higher in the ALC group than in the HBV group (**Figures 6E, F**). In summary, the lower number of intrahepatic T cells in the ALC group may be regulated by Galectin-9 secreted by M1 cells.

### 3.4 The characteristics of intrahepatic B cells associated with ALC and HBV-related cirrhosis

**Figure 7:**
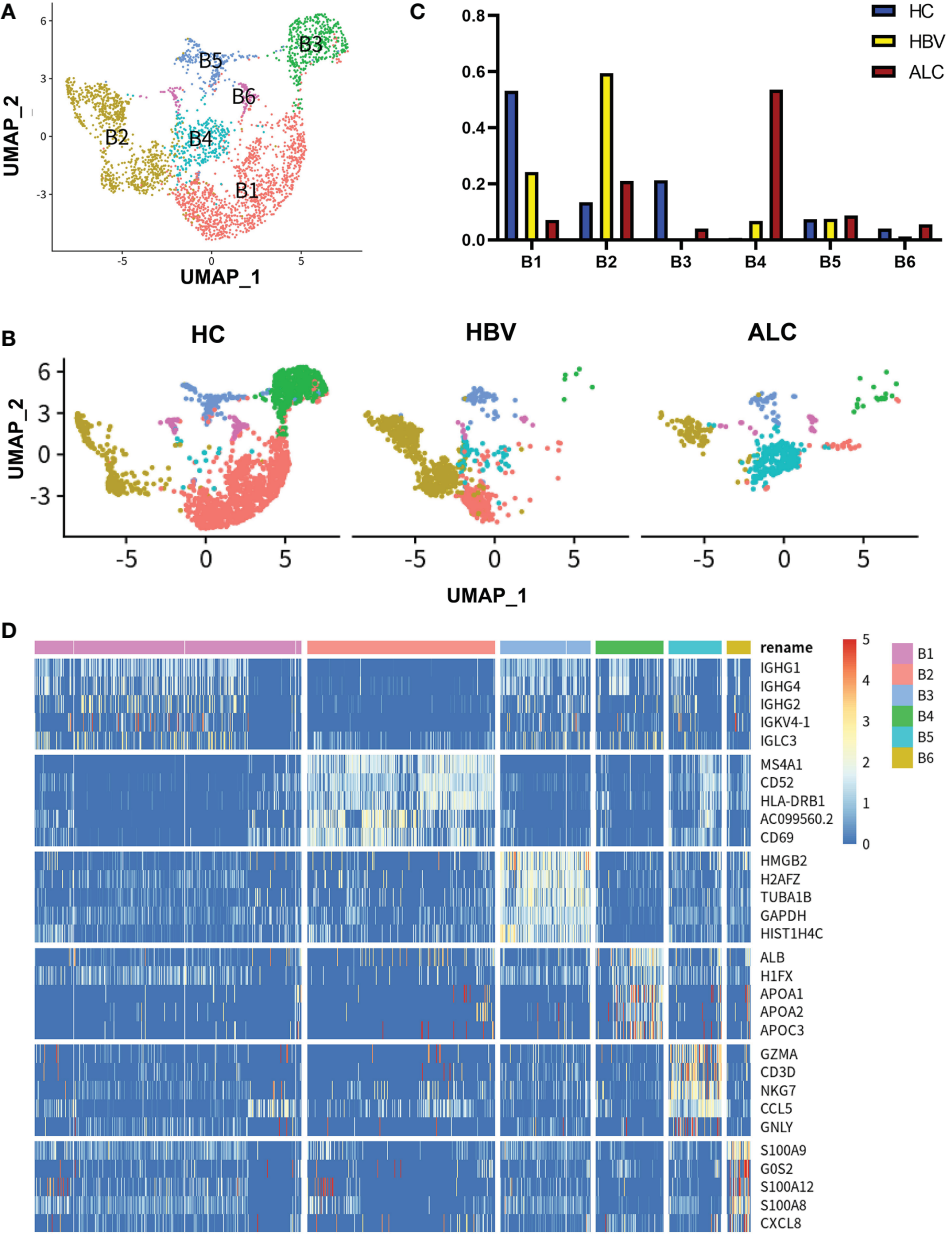
scRNA-seq identified the characteristics of B cells among three groups. **(A)** A total of 3,233 B cells from the HC, ALC, and HBV groups were classified into B1–B6 clusters. **(B)** The distribution of the B1–B6 clusters in each group was shown using a UMAP plotting. **(C)** The proportion of the B1–B6 clusters in the HC, HBV and ALC groups. **(D)** High-expression genes of the B1–B6 clusters were presented using a heatmap.

**Figure 8:**
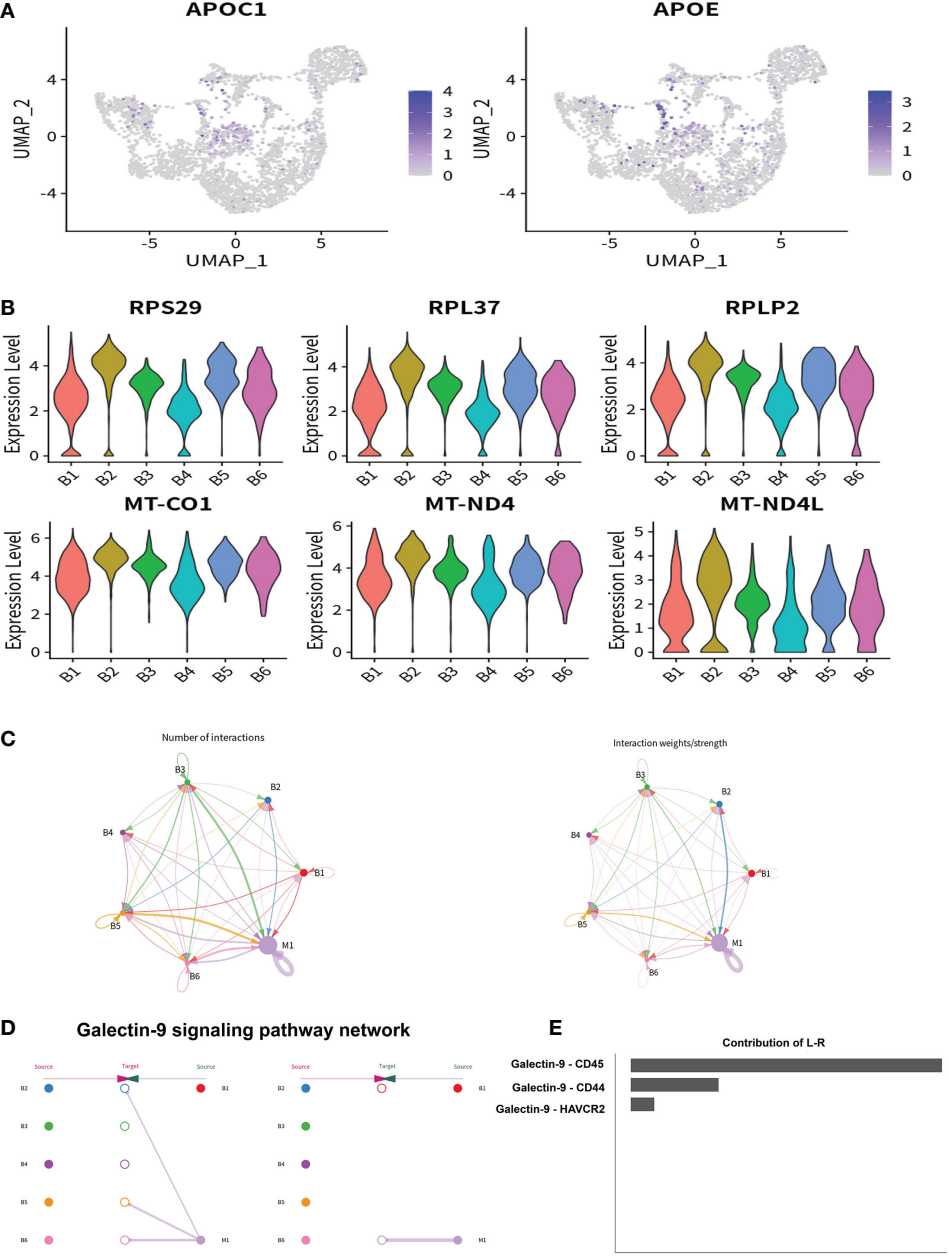
The potential regulatory pathway between M1 and B cells. **(A)** The expression of lipid metabolism related genes in B1–B6 clusters were shown using a UMAP plotting. **(B)** The expression of ribosome and mitochondrial related genes in B1–B6 clusters were shown using a violin plotting. **(C)** The Ligand/receptor analysis between the M1 and B1–B6 clusters. **(D)** Galectin-9 was identified as a potential signaling pathway and Galectin-9 was mainly originated from the M1 cluster. **(E)** Galectin-9-CD45 was the mainly Ligand-receptor pathway.

To identify the characteristics of intrahepatic B cells associated with ALC and HBV-related cirrhosis, 3,233 B cells (CD79A^+^CD79B^+^) were analyzed and annotated into six clusters (B1–B6, **Figure 7A**). The proportions of the B1–B6 clusters differed significantly among the three groups (**Figures 7B, C**) and the B4 cluster ratio was significantly higher in the ALC group than in the HBV group. The heatmap showed that the B4 cluster expressed a series of lipid-metabolism-associated genes, such as APOA1, APOA2, and APOA3 (**Figure 7D**), and UMAP plotting also showed that B4 cells highly expressed APOC1 and APOE (**Figure 8A**), implying that the B4 cluster may be associated with steatosis in the ALC group. The expression of ribosome and mitochondria RNA were significantly lower in the B4 cluster than in the other clusters (**Figure 8B**), indicating that cells in the B4 cluster were in an inactive status. In addition, plasma cells (B1 cluster), which expressed IGHG1 and IGHG2 (**Figure S7**), were significantly lower in the ALC group (**Figure 7C**). Finally, Ligand/receptor analysis of the M1 and B cells was conducted to determine the mechanism for the reduced B cell numbers. As shown in **Figure 8C**, multiple interactions were found, and the Galectin-9 pathway was also discovered in M1-B cells Ligand/receptor analysis (**Figure 8D**), which was also affected by Galectin-9-CD45 Ligand-receptor (**Figure 8E**).

## 4 Discussion

Liver cirrhosis, initiated by alcohol abuse or HBV infection, has placed a tremendous burden on the healthcare system with limited treatment options (1, 2). Once decompensation occurs, the progression to ACLF often leads to death. Thus, new and effective methods are urgently needed to reduce the incidence of alcoholic and HBV-related liver cirrhosis. Studies indicate that immune cells play an important role in ALC and HBV-related cirrhosis (10, 20–22). The current study used scRNA-seq to compare the immune status of liver tissue from patients with alcohol and HBV-related cirrhosis. ALC patients were shown to exhibit different immune characteristics than those in the HBV group, including an increased ratio of intrahepatic monocyte/macrophages and a decreased ratio of intrahepatic T and B cells. Ligand/receptor analysis indicated that Galectin-9 expressed on KCs may contribute to reduced T and B cell levels in the ALC liver, suggesting that this factor could serve as a potential therapeutic target.

The management and treatment of HBV-related cirrhosis are well established (23), but limited progress has been made in developing treatments for ALC. Immune cells are shown to play an important role during alcohol-induced liver injury and cirrhosis (20–22). Thus, comparing the immune characteristics of ALC and HBV-related cirrhosis could provide additional insights into the treatment of ALC. The current study identified an increased ratio of intrahepatic monocyte/macrophages and a decreased ratio of intrahepatic T and B cells in the ALC group than in the HBV group. IHC staining for CD68, CD3, and CD19 confirmed the scRNA-seq data, indicating that the immune status was altered in the ALC group. Further analysis showed that the increased monocyte/macrophages in ALC was mainly CD5L^+^ KCs. Study showed that CD5L directly regulated the switch of the pathogenic Th17 cells to non- pathogenic Th17 cells (24), which were reported as the critical disease-causing cells in alcoholic liver disease (25–27). CD5L also inhibits immune cell infiltration during CCL4-induced chronic liver injury, reducing injury and inflammation in a mouse model (28). In the current study, the M1 cluster expressed metabolism-associated genes, such as CETP, LIPA, and APOE, suggesting that a potential interactant between M1 cluster and hepatic steatosis in ALC and HBV-related cirrhosis. Alcohol is a direct cause of fatty liver disease and steatohepatitis (29) and the downstream metabolites of alcohol-acetaldehyde impair hepatic mitochondrial functions such as fatty acid oxidation, resulting in lipid accumulation (30). In our study, the H&E staining definitely showed that ALC group had severe steatosis compared with HBV group. Previous studies have suggested that lipid accumulation in the liver causes macrophage-induced inflammation and necrosis (31–33). These findings together suggest that the M1 cluster may contribute to ALC pathogenesis.

Several studies have shown that innate immunity plays an important role in the pathogenesis of alcoholic liver disease, while adaptive immunity has received less attention (34). Thus, this study explored the status of adaptive immunity in ALC. The results showed a decreased ratio of intrahepatic T and B cells. While altered peripheral T cell numbers are associated with alcohol abuse (35, 36), little is known about the role of T cells in alcoholic cirrhosis. B cells are also shown to be lower in heavy alcoholics than in moderate or light drinkers and the loss of circulating B cells is particularly severe in individuals with alcoholic liver disease (37, 38), which is consistent with the current study. In our study, high-expression gene enrichment analysis showed that the M1 cluster was involved in negatively regulating immune processes. To identify the mechanism for the T and B cell exhaustion, ligand/receptor analysis was performed between M1 and T or B cells. Interestingly, the Galectin-9 signaling pathway was involved in both M1-T cell and M1-B cell interactions. Galectin-9 is a β-galactoside binding lectin that plays an immunomodulatory role in various microbial infections (39). This factor is an important regulator of T-cell immunity that induces apoptosis in specific T-cell subpopulations associated with autoimmunity and inflammation (40). Many evidences had proved the immune regulatory function of Galectin-9. Specifically, Galectin-9 is associated with the expansion of regulatory T cells, the contraction of CD4^+^ effector T cells, and the apoptosis of HCV-specific cytotoxic T lymphocytes (41). Zhu et al. found that the administration of Galectin-9 *in vivo* resulted in the selective loss of interferon-gamma-producing cells and the suppression of Th1 autoimmunity (42). Moreover, Galectin-9 is also shown to directly suppress activated B cells (43, 44) and attenuate BCR activation and signaling (44). The results from the current study indicated that Galectin-9 was primarily originated from KCs (the M1 cluster), and IHC staining showed that Galectin-9 was significantly higher in the ALC group. It is probable that Galectin-9 expressed on KCs led to T and B cells reduction and ultimately impaired the adaptive immune response in ALC patients. Thus, Galectin-9 could serve as a potential therapeutic target in ALC.

In our study, the B4 cluster ratio was significantly higher in the ALC group than in the HBV group. Re-cluster analysis and annotation analysis showed that lipid-metabolism-associated genes were highly expressed in B4 cells, implying that the B4 cluster was associated with steatosis in the ALC group. Meanwhile, we also found that plasma cells (the B1 cluster) were also significantly reduced in the ALC group, indicating that the humoral immune response may be impaired. Those results were a further proof of the impaired adaptive immunity in ALC.

The current study still has some limitations. On one hand, due to the high requirements of scRNA-seq on specimen quality and cells activity, only a few human liver samples had been included in this research. Further studies using larger and more heterogeneous human liver samples are needed to verify the results. On the other hand, the study only investigated the correlation between Galectin-9 and ALC, with insufficient evidence to prove its causality. Genes knockout and functional research are need to in-depth explore the role of galectin-9 in ALC.

In conclusion, this study is the first to compare the immune status, including innate and adaptive immune cell function, between patients with alcoholic and HBV-related liver cirrhosis using scRNA-seq. ALC patients exhibited different immune characteristics with HBV group, including an increased ratio of intrahepatic monocyte/macrophages and a dysfunctional adaptive immune response in the liver. Galectin-9 expressed on KCs may be associated with reduced T and B cells and could serve as a potential therapeutic target for ALC patients.

## Data availability statement

The datasets presented in this study can be found in online repositories. The names of the repository/repositories and accession number(s) can be found in the article/**Supplementary Material**.

## Ethics statement

This study was approved by the Ethics Committee of The Third Xiangya Hospital Central South University (No:2020-S024). The patients/participants provided their written informed consent to participate in this study.

## Author contributions

PZ and YM planned the study. PZ and HL wrote the first draft of the manuscript. BP and KC collected the clinical data. All authors contributed to the design and interpretation of the study and reviewed the final draft of the manuscript. All authors contributed to the article and approved the submitted version.
