# Supplementary material for: Single-cell RNA transcriptomics reveals differences in the immune status of alcoholic and hepatitis B virus-related liver cirrhosis
Source: Front Endocrinol (Lausanne). 2023 Feb 2;14:1132085. doi: 10.3389/fendo.2023.1132085 (PMC9932584; doi:10.3389/fendo.2023.1132085)
Supplement: Supplementary file 1 [file DataSheet_1.docx]

Supplementary Material


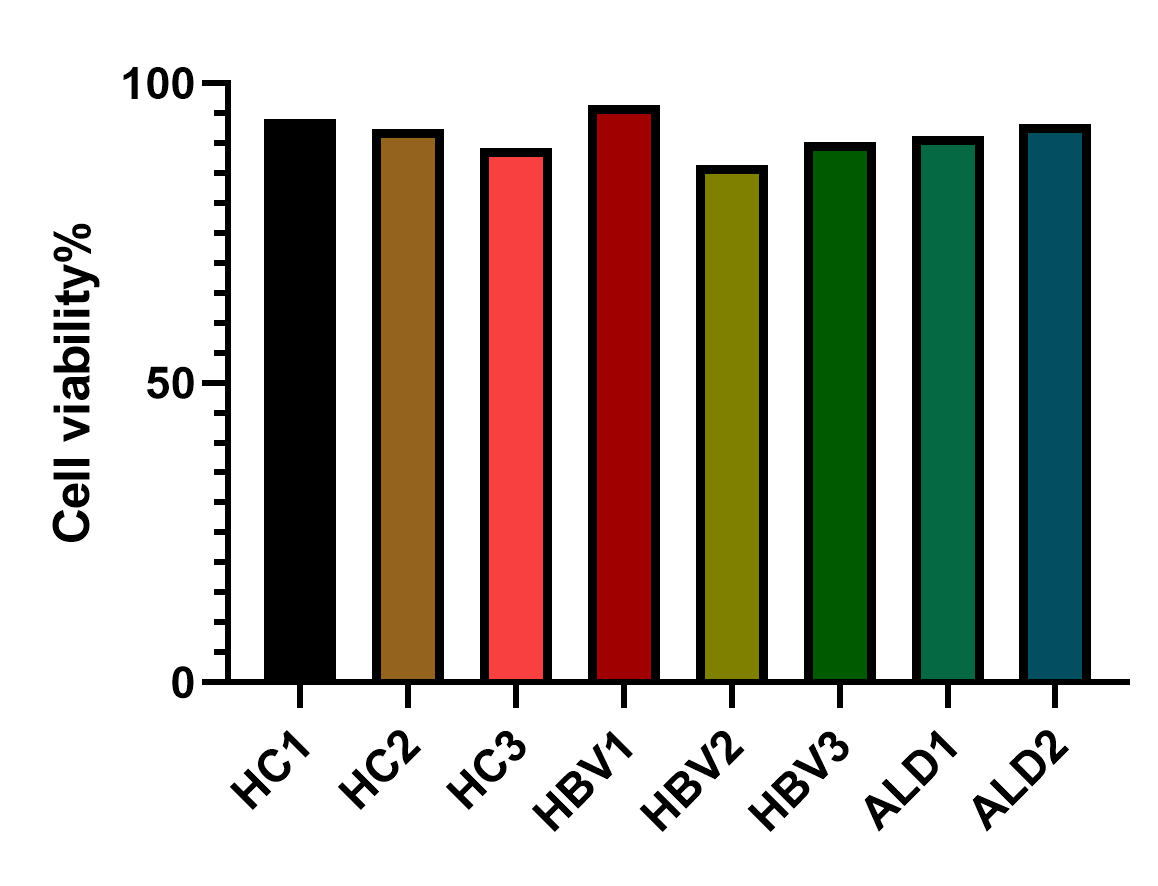


**Figure S1 Cell viability of each sample.**


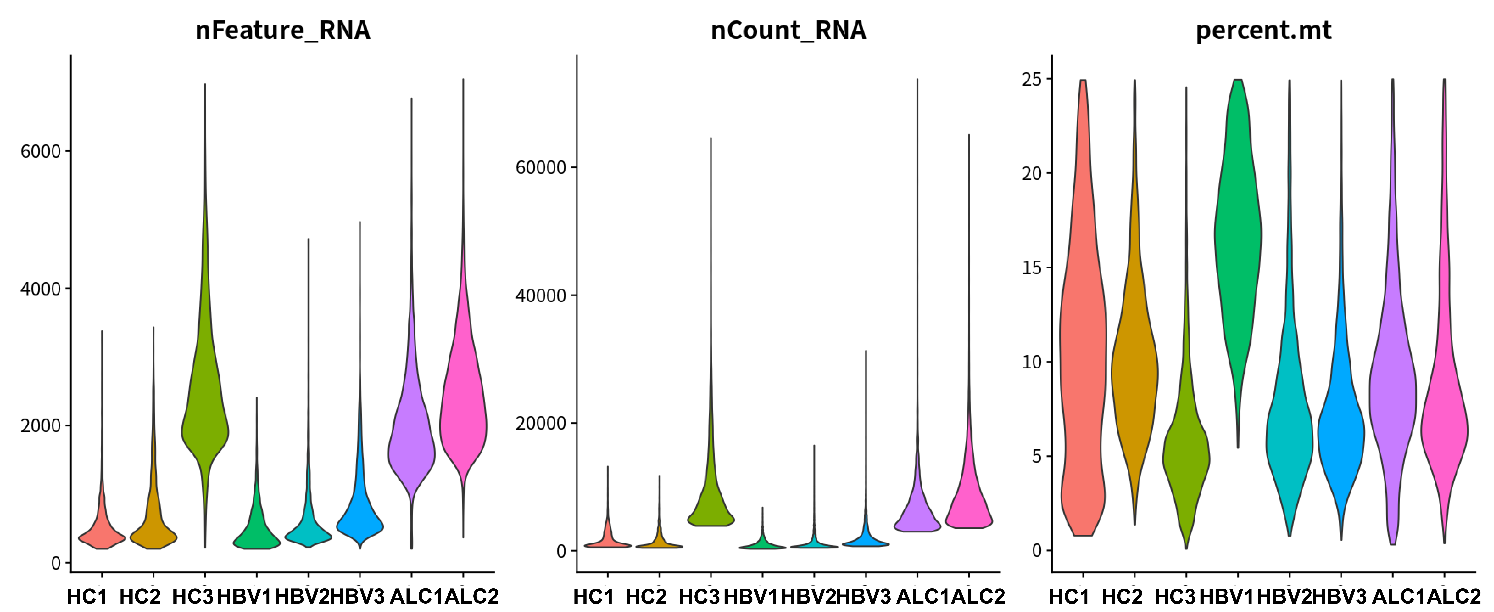


**Figure S2 Quality control data of scRNA-seq in our study.**


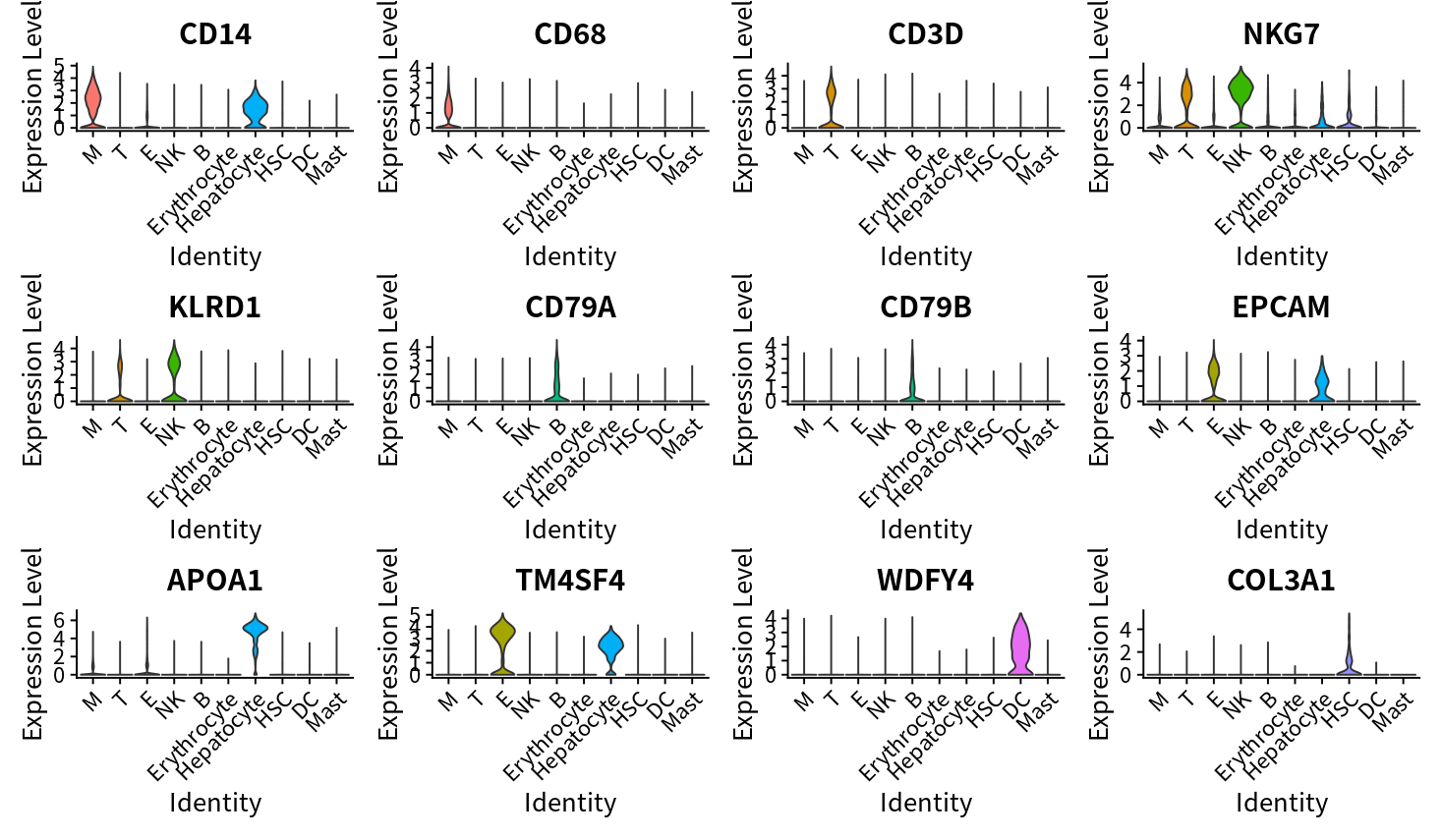


**Figure S3 The classical marker genes for each cluster in violin plotting.**


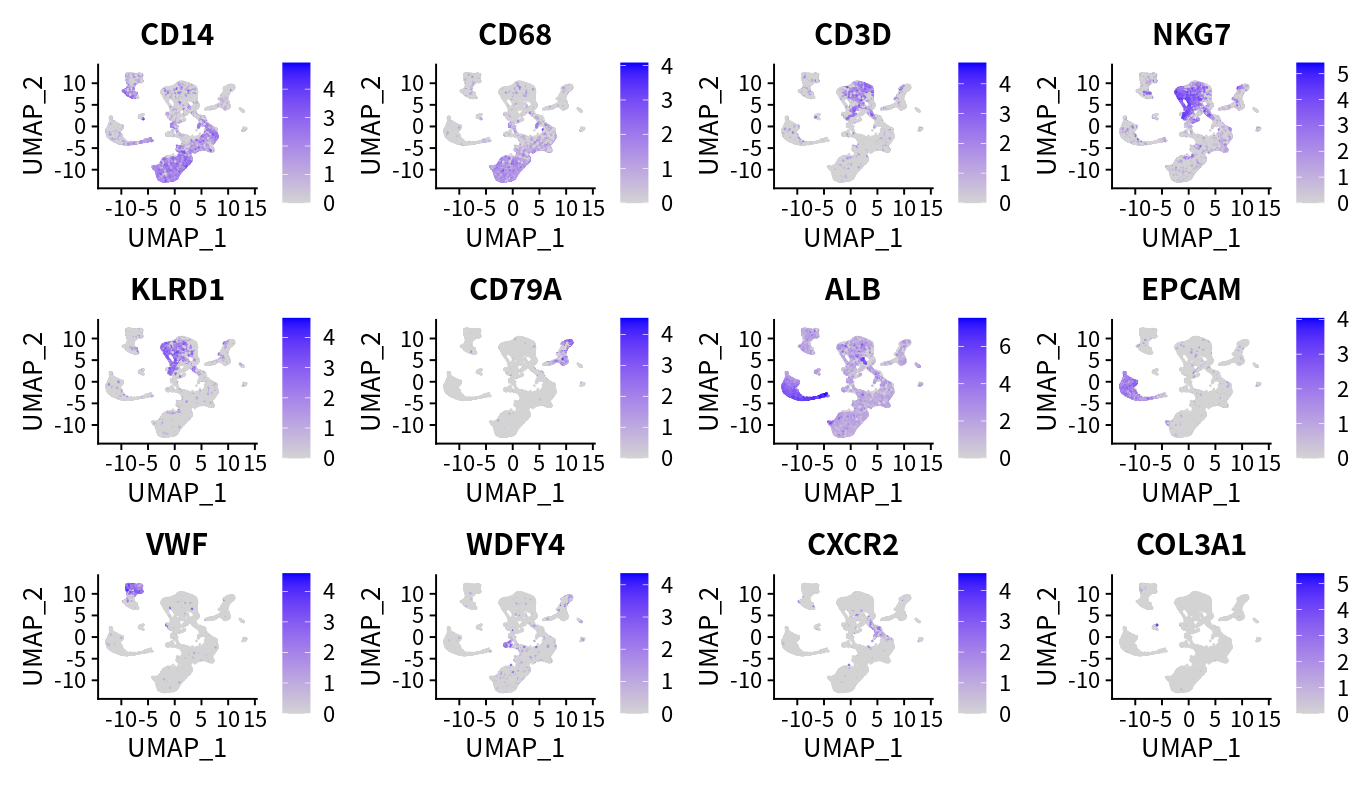


**Figure S4 The classical marker genes for each cluster in UMAP plotting.**


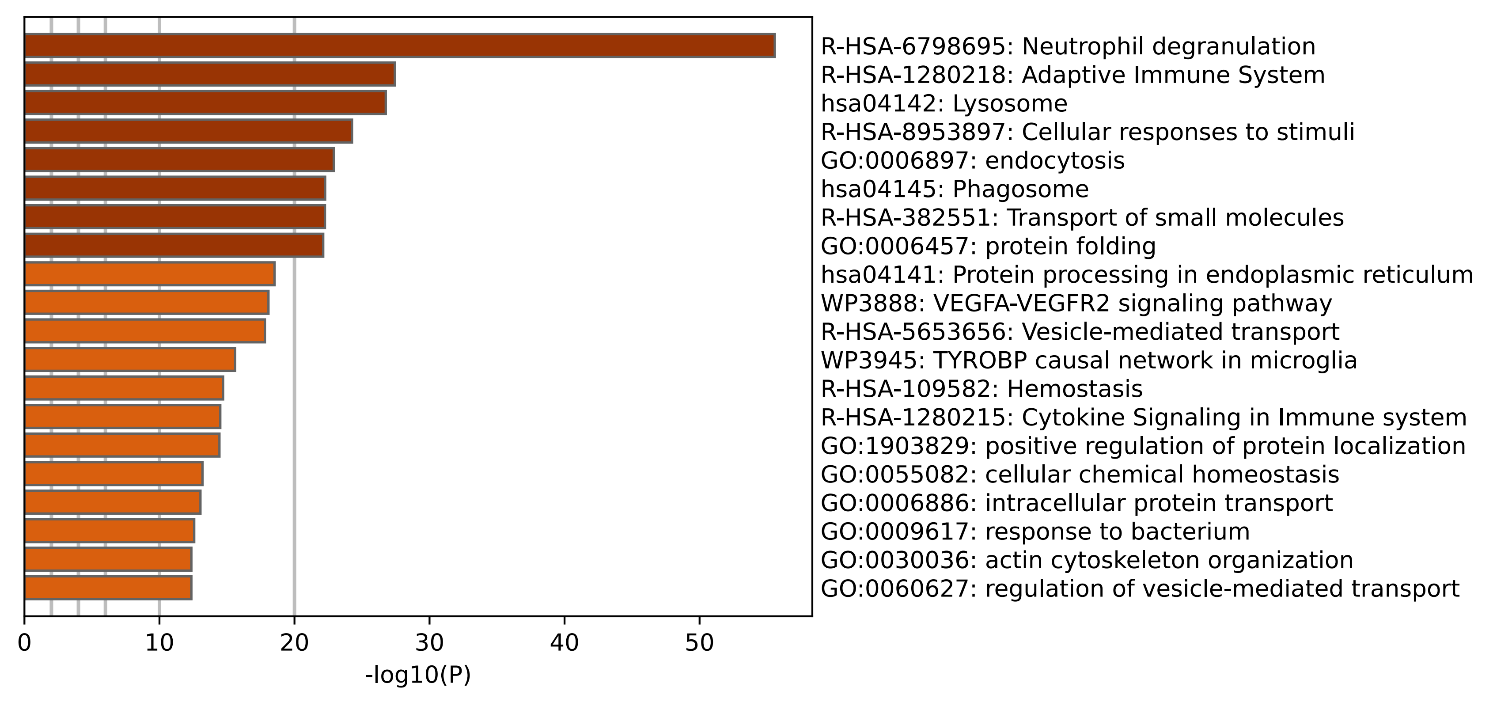


**Figure S5 High expression genes enrichment analysis for M4 clusters.**


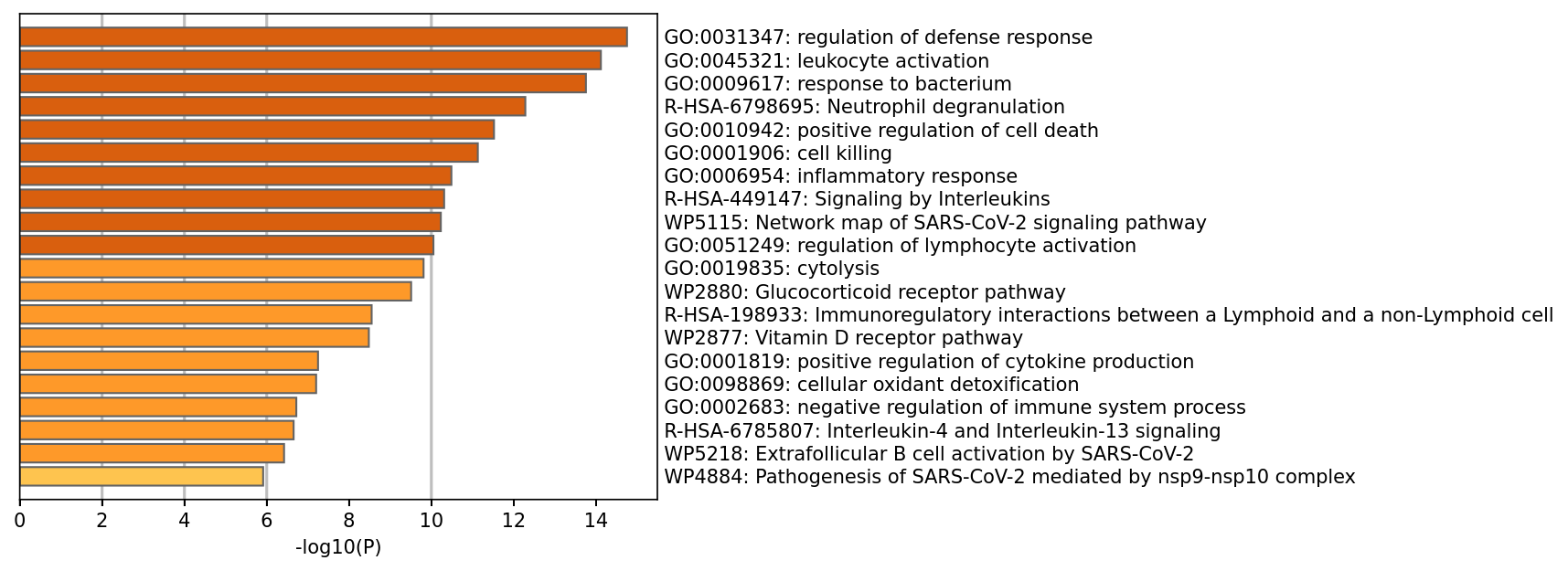


**Figure S6 High expression genes enrichment analysis for M5 clusters.**


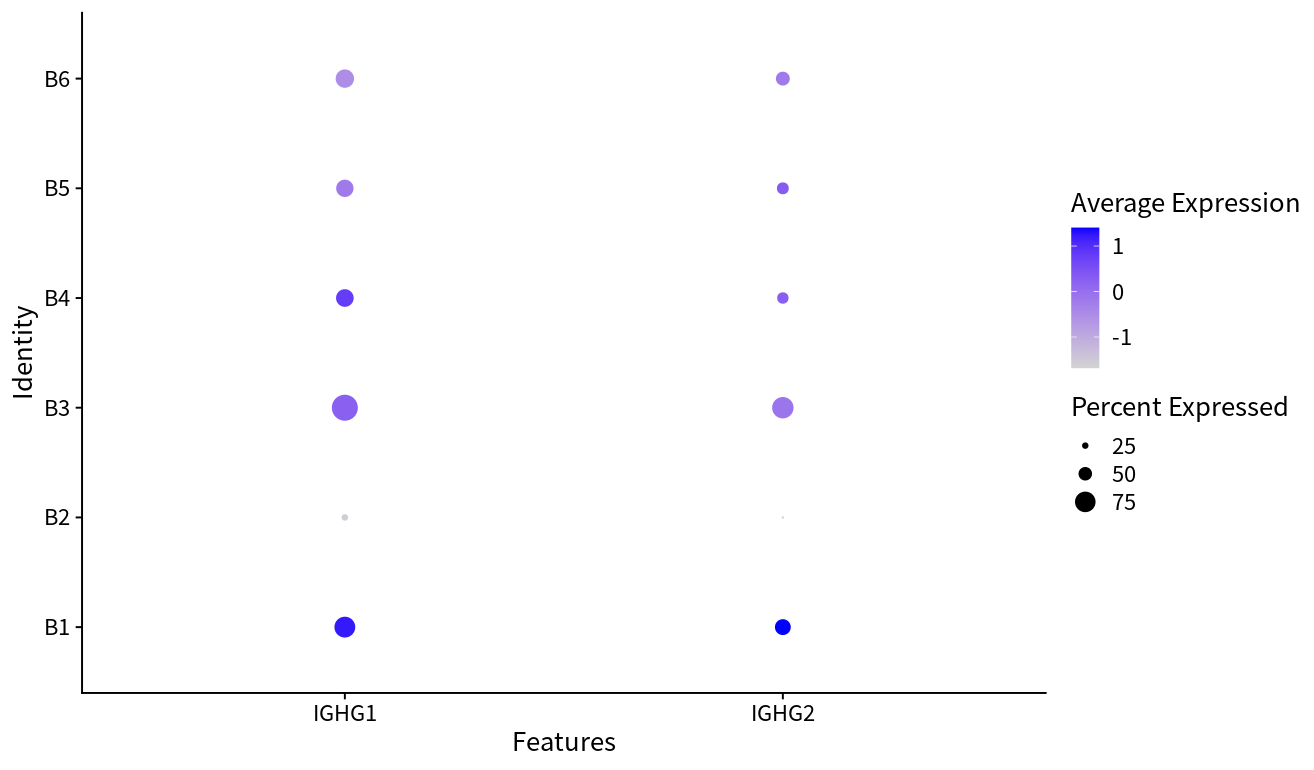


**Figure S7 The expression of IGHG1 and IGHG2 for B1-B5 cluster.**
